# Comparison of prognostication by IPSS-M, IPSS-R and AIPSS-MDS in the context of limited availability of molecular data in daily clinical practice

**DOI:** 10.1007/s00277-025-06570-0

**Published:** 2025-09-06

**Authors:** Felicitas Schulz, Carolin Kellersmann, Beate Betz, Barbara Hildebrandt, Annika Kasprzak, Corinna Strupp, Felicitas Thol, Michael Heuser, Christina Ganster, Fabian Beier, Katja Sockel, Wolf-Karsten Hofmann, Andrea Kuendgen, Paul Jaeger, Michael Pfeilstoecker, Michael Lauseker, Sascha Dietrich, Nobert Gattermann, Kathrin Nachtkamp, Detlef Haase, Ulrich Germing

**Affiliations:** 1 Department of Hematology, Oncology and Clinical Immunology, Heinrich-Heine-University Düsseldorf, Moorenstr. 5, 40225 Düsseldorf, Germany; 2 Department of Human Genetics, Heinrich-Heine-University Düsseldorf, Düsseldorf, Germany; 3 Department of Hematology, Hemostasis, Oncology and Stem Cell Transplantation, Hannover Medical School, Hannover, Germany; 4 Department of Hematology and Medical Oncology, INDIGHO laboratory University Medical Center Göttingen (UMG), Göttingen, Germany; 5 Department of Hematology, Oncology, Hemostaseology and Stem Cell Transplantation, Medical Faculty, RWTH Aachen University, Aachen, Germany; 6 Medical Clinic and Policlinic I, University Hospital Carl Gustav Carus Dresden, Dresden, Germany; 7 Department of Hematology and Oncology, Medical Faculty Mannheim, Heidelberg University, Mannheim, Germany; 8 3rd Medical Department for Hematology and Oncology, Hanusch Hospital, Vienna, Austria; 9 Institute for Medical Information Processing, Biometry, and Epidemiology, Faculty of Medicine, LMU Munich, Munich, Germany

**Keywords:** Myelodysplastic syndromes, MDS, Prognostication, IPSS

## Abstract

The IPSS-M was developed to revolutionize the prediction of MDS patients’ survival by incorporating molecular data. To compensate for lack of access to molecular analyses, the AIPSS-MDS, a supervised machine learning algorithm exclusively based on clinical and cytogenetic data, was developed by the Spanish MDS Group. We used data of the Düsseldorf MDS Registry and included 207 of more than 8500 registry patients whose IPSS-M-requested complete molecular data were known to compare and validate prognostication regarding OS and LFS of the IPSS-M, IPSS-R and AIPSS-MDS. All three tools reliably prognosticated median OS of patients even in a comparatively small patient cohort. The IPSS-M provided the most accurate prediction of median OS while the frequent lack of molecular data persists as an obstacle in daily clinical practice. Due to these circumstances, the IPSS-R remains the prognostication tool with the widest applicability. Based on our data, prognostication using the AIPSS-MDS is also feasible but less precise.

## Introduction

Myelodysplastic syndromes (MDS) comprise clonal disorders of the hematopoietic stem cell characterized by dysplasia of the bone marrow and the increased risk of transforming into acute myeloid leukemia (AML) [1]. Patients present with different values of hematopoietic insufficiency meaning pancytopenia as worst-case scenario with the frequent need of transfusion therapy and the permanent risk of infectious complications. Therefore, prognosis and treatment options differ widely between patients, making precise prognostication even more important [2]. 

Since 2022, according to the 5th edition of the WHO classification, the terminology has changed from myelodysplastic syndromes to myelodysplastic neoplasms thereby emphasizing the underlying neoplastic nature [3]. Classification is now subdivided into ‘MDS with defining genetic abnormalities’ and ‘MDS, morphologically defined’ with three subcategories each, incorporating molecular data to a greater extent. This progression suits the enhancement of the well-known revised international prognostic scoring system (IPSS-R) to the molecular international prognostic scoring system (IPSS-M). While the IPSS-R, a well-established prognostication tool since 2012, relies on five hematologic and cytogenetic features (hemoglobin, absolute neutrophil and platelet count, bone marrow blasts and cytogenetic risk category) assigning patients to five different risk groups, prognostication with the IPSS-M is based on molecular genetics as well to revolutionize the prediction of MDS patients’ survival by incorporating molecular data [4, 5]. Known mutation status of 31 genes assigns patients to six different risk groups with different probability of overall survival (OS) and leukemia free survival (LFS).

To compensate for complexity and inaccessibility of molecular analyses, the AIPSS-MDS (Artificial Intelligence Prognostic Scoring System for MDS), a supervised machine learning algorithm exclusively based on clinical and cytogenetic data, was developed by the Spanish MDS Group [6]. Including 8 variables (age, gender, hemoglobin, leukocyte and platelet count, neutrophil percentage, bone marrow blasts and cytogenetic risk group), the AIPSS-MDS achieved superior accuracy in predicting OS and LFS in patients of the Spanish Group of Myelodysplastic Syndromes compared to the (age-adjusted) IPSS-R using the machine learning technique random survival forests [6].

To compare and validate these three prognostication tools and put them into context of clinical daily practice we used data of the Düsseldorf MDS Registry.

## Methods

**Fig. 1:**
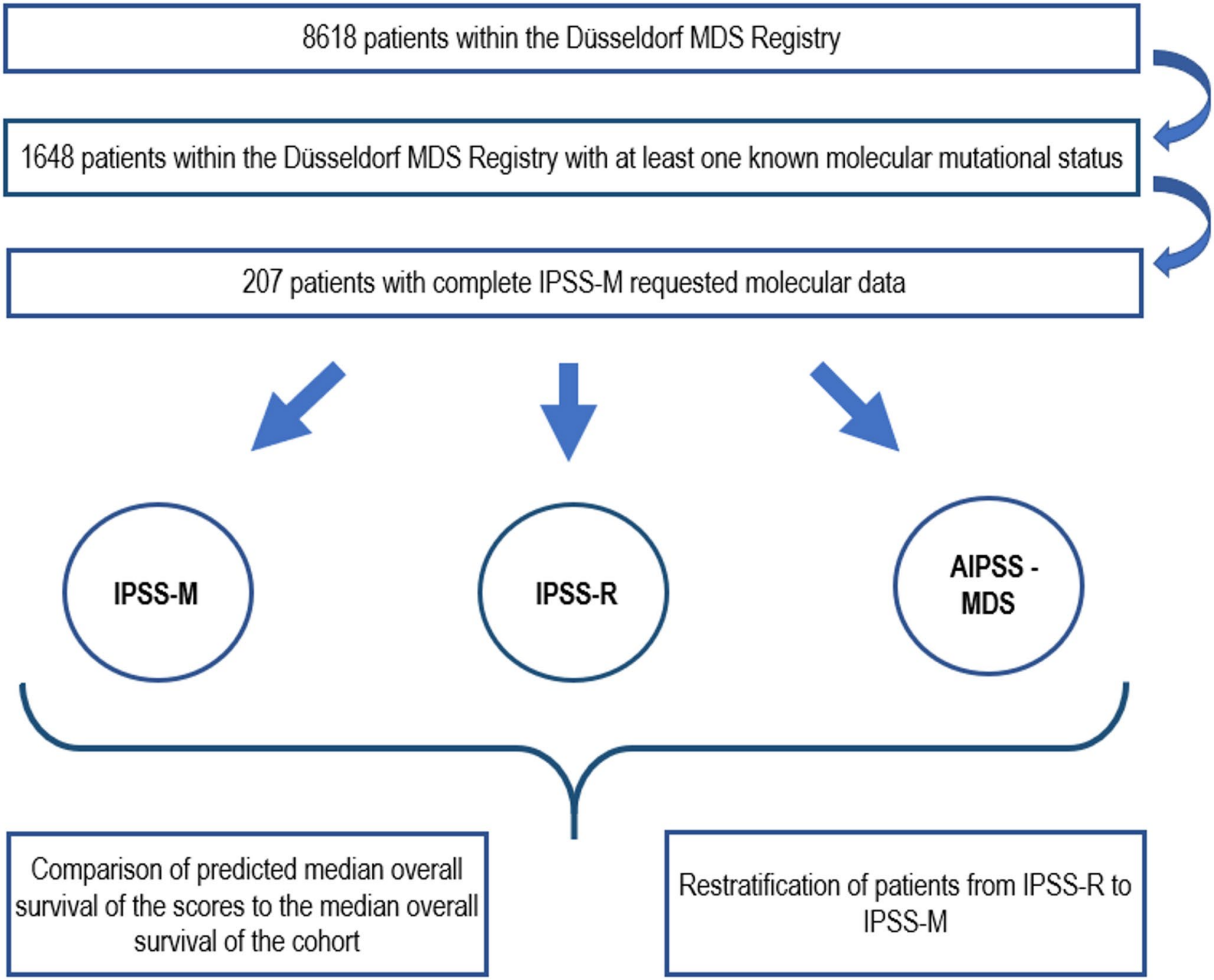
Visualized concept of the analyses

Data was taken from the Düsseldorf MDS Registry, including patients from Germany and Austria, which, at the time of the analyses, contained more than 8500 patients with diagnosed MDS. The analysis was conducted in accordance with the Declaration of Helsinki and approved by the Ethics Committee of the Heinrich-Heine University in Duesseldorf. Informed consent was obtained from all subjects in the study. All patients were classified according to WHO classification of 2016. Follow-ups were done until death or patients’ last visit. For patients who underwent allogeneic stem cell transplantation, the date of the transplantation was set as last follow up. Of 1648 patients from the Registry with at least one known molecular mutational status available, 207 patients offered all required parameters and IPSS-M requested molecular data. We used these 207 patients (59% male, median age 62 years) and their disease characteristics from time of diagnosis to assess their prognosis based on the IPSS-R, IPSS-M and AIPSS-MDS. To prevent from statistical uncertainty of the IPSS-M, the remaining 1441 patients whose molecular data were incomplete were excluded. Detailed patient demographics are shown in Table 1. Using Kaplan-Meier based survival analysis, we determined the median overall survival of each patient group within the categories of IPSS-M and IPSS-R and compared it to the predicted median OS of the three prognostication tools. We also calculated the predicted median OS by AIPSS-MDS and compared it to the results of IPSS-M and IPSS-R. Following the methods of the original publication of Mosquera Orgueira et al., we divided the patients according to AIPSS-MDS results into equal quintiles and assessed OS and LFS using Kaplan-Meier based survival analysis as well. To assess the discrimination of the prognostic models, we calculated the c-indices of the IPSS-R and IPSS-M but decided to not compare them to the c-index of the AIPSS-MDS quintiles as these mean artificially implemented subgroups of homogenous sizes in contrast to the IPSS-R and IPSS-M groups because the AIPSS-MDS does not form groups by itself but refers to an individual OS for each patient. Furthermore, we did a cross-table calculation to re-stratify patients from IPSS-R to IPSS-M. The detailed concept of the analyses is illustrated in Fig. 1. Compared to the IPSS-R with cytogenetics as the most influential variable followed by marrow blast count and hemoglobin level, the IPSS-M focuses on cytogenetics and molecular genetics as most important factors. In contrast, the risk stratification of the AIPSS-MDS, mainly developed for those without access to advanced genomic tools, has the marrow blast count and hemoglobin level as most potent variables followed by age and cytogenetics. Impact of the different variables included in the three prognostication tools is shown in Table 2.

**Table 1:** Detailed patient demographics

	*n* = 207
Gender, n (%)) male female	121 (58.5) 86 (41.5)
Age in years, median (range)	69 (20–88)
White blood cell count per nl, median (range)	4.1 (0.4–52.0)
Hemoglobin in g/dL, median (range)	9.9 (5.1–14.8)
Platelets per nl, median (range)	128 (6–1.4)
Absolute neutrophil count per nl, median (range)	2.3 (0.4–32.4)
Marrow blast count, median (range)	3 (0–19)
2022 WHO categories, n (%) MDS-LB MDS-LB-RS MDS-IB1 MDS-IB2 MDS-TP53 MDS-5q MDS-SF3B1 MD-CMML MP-CMML	78 (37.6) 8 (3.9) 21 (10.1) 18 (8.7) 9 (4.3) 22 (10.6) 28 (13.5) 17 (8.2) 6 (2.9)
Cytogenetic risk category very good good intermediate poor very poor	8 (3.9) 136 (65.7) 31 (15.0) 17 (8.2) 15 (7.2)
Molecular genetics TP53 MLL PTD FLT3-ITD or TKD ASXL1 CBL DNMT3A ETV6 EZH2 IDH2 KRAS NPM1 NRAS RUNX1 SF3B1 SRSF2 U2AF1 BCOR BCOR1L CEBPA ETNK1 GATA2 GNB1 IDH1 NF1 PHF6 PPM1D PRPF8 PTPN11 SETBP1 STAG2 WT1	23 (11.1) 3 (1.4) 2 (1.0) 39 (18.8) 5 (2.4) 24 (11.6) 6 (2.9) 13 (6.3) 3 (1.4) 2 (1.0) 1 (0.5) 5 (2.4) 27 (13.0) 37 (17.9) 21 (10.1) 10 (4.8) 5 (2.4) 3 (1.4) 3 (1.4) 0 1 (0.5) 0 7 (3.4) 0 3 (1.4) 0 0 1 (0.5) 7 (3.4) 1 (0.5) 24 (11.6)
Treatment, n (%) No treatment/BSC Allografting Hypomethylating agents Lenalidomid Low-dose chemo Autologous SCT	116 (56.0) 47 (22.7) 23 (11.1) 13 (6.3) 5 (2.4) 3 (1.5)
AML transformation, n (%)	49 (23.7)
Median overall survival, months	33 (1–225)
Death, n (%)	91 (44.0)

**Table 2:** Included variables and their impact within the three different prognostication tools (larger and bold font meaning higher impact)

IPSS-*R*	Hb	ANC	Plt	Marrow blasts	Cytogenetics				
*IPSS-M*	**Hb**		Plt	**Marrow blasts**	**Cytogenetics**	**Molecular genetics**			
*AIPSS-MDS*	**Hb**	RNC	Plt	**Marrow blasts**	**Cytogenetics**		**Age**	Gender	WBC
ANC: absolute neutrophil count, Hb: hemoglobin, RNC: relative neutrophil count, WBC: white blood cells									

## Results

**Fig. 2:**
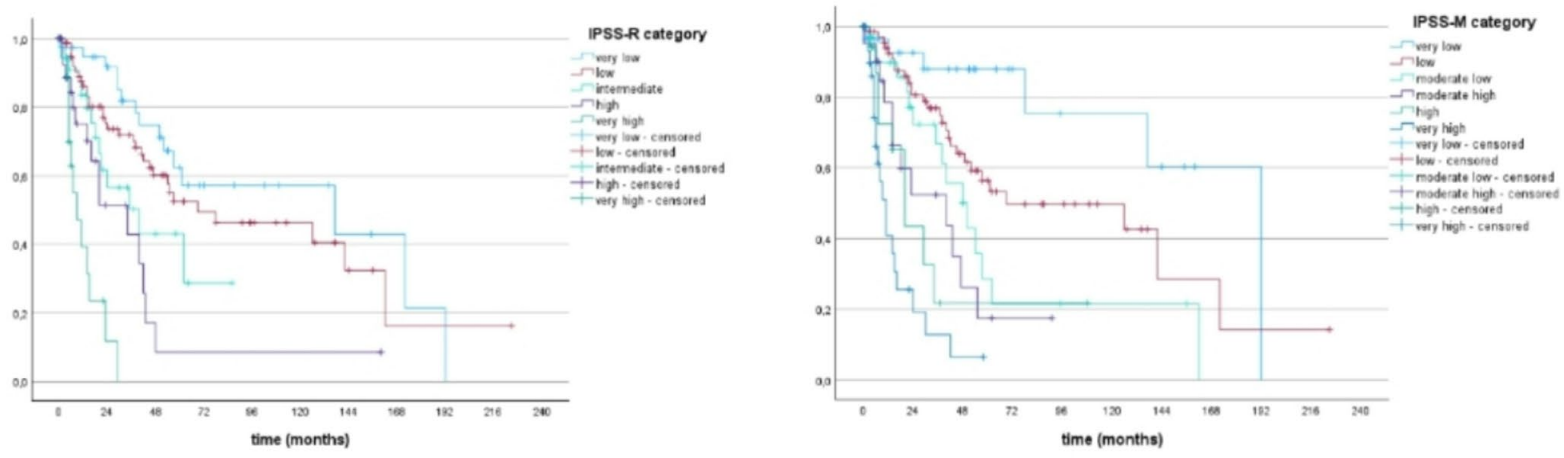
Overall survival of patients according to IPSS-R and IPSS-M risk groups

**Fig. 3:**
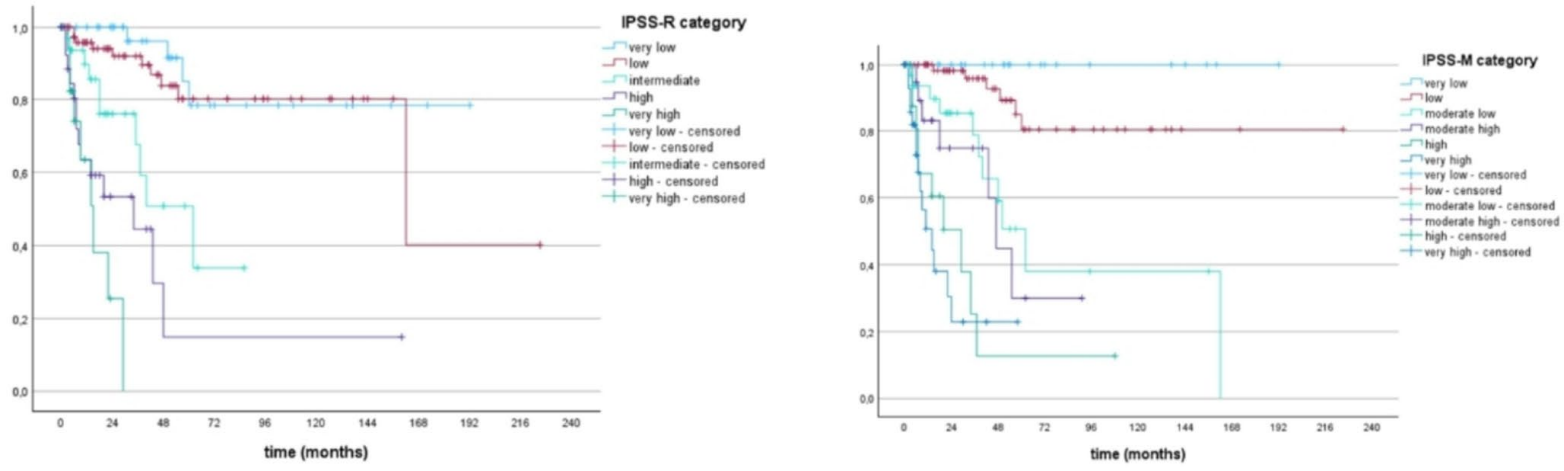
Leukemia-free survival according to IPSS-R and IPSS-M risk groups

**Fig. 4:**
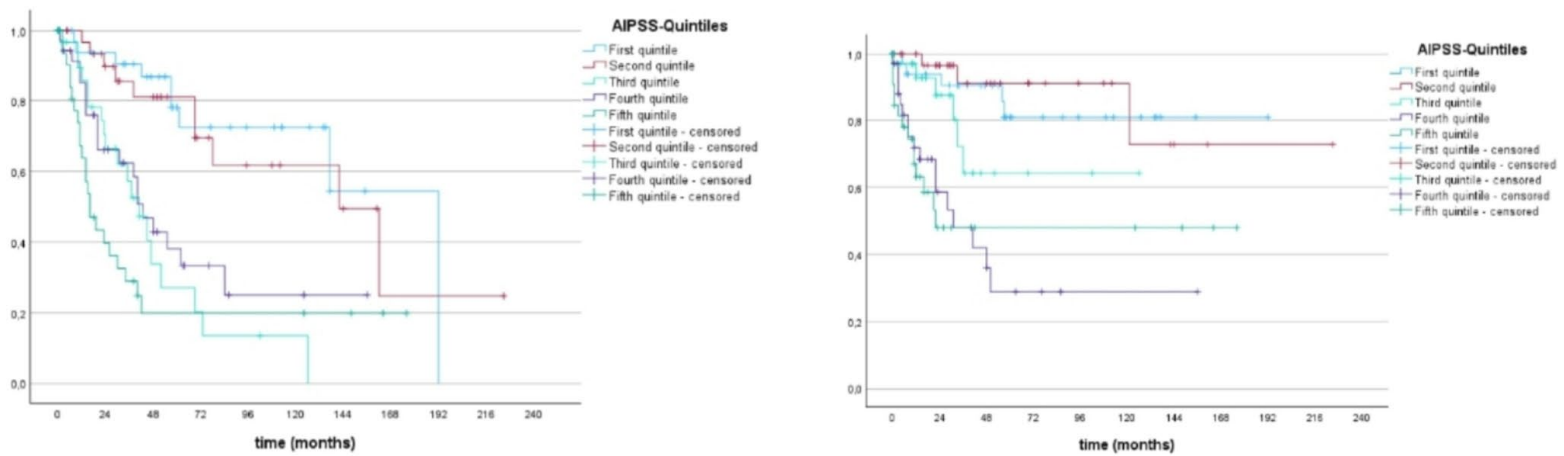
Overall survival (left) and Leukemia-free survival (right) of patients according to AIPSS-MDS quintiles

**Fig. 5:**
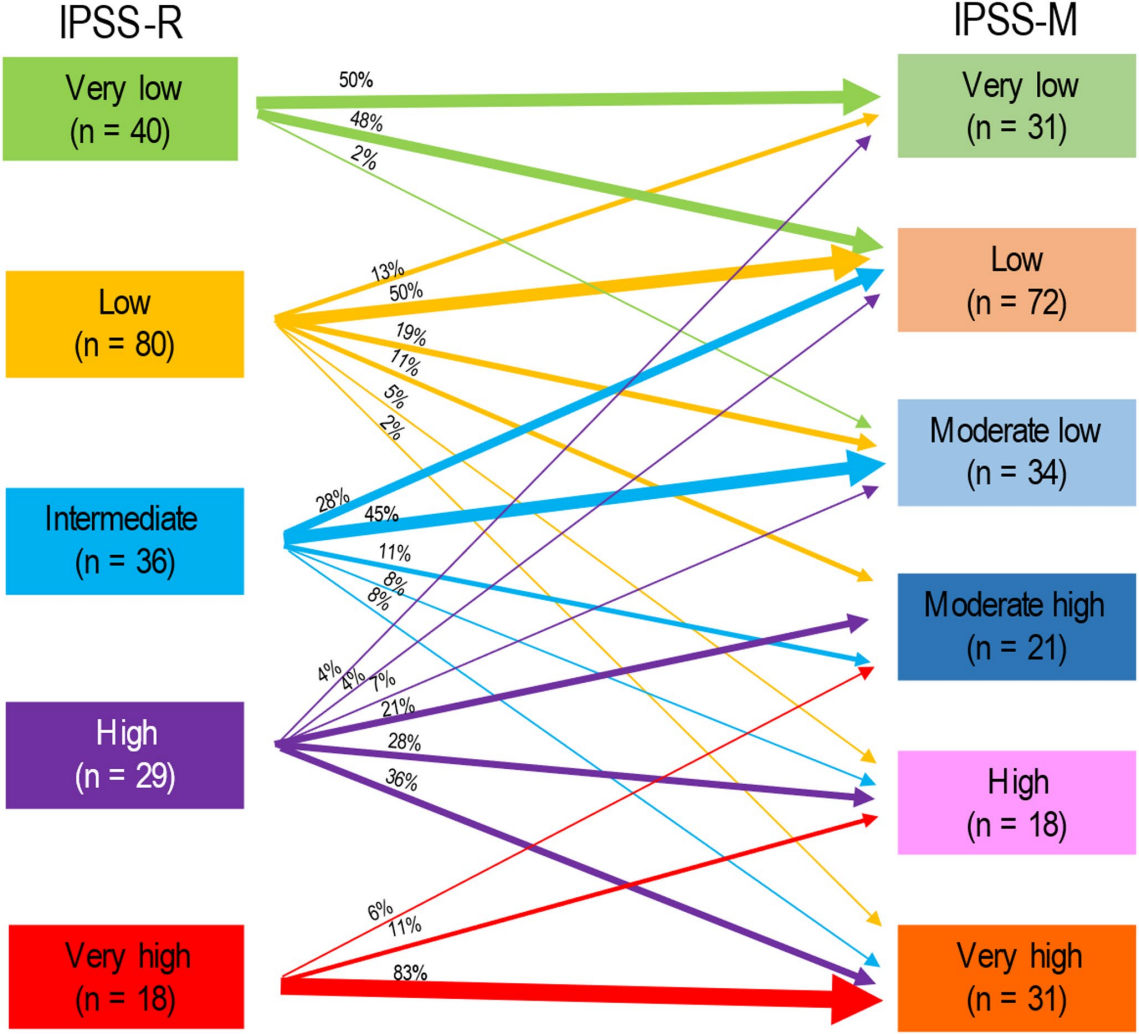
Re-stratification of patients from IPSS-R to IPSS-M

Of more than 8600 patients within the Düsseldorf MDS Registry, 1648 patients had at least one known molecular mutational status. Of these 1648 patients, only 207 (12.6%) could be included into our analyses due to missing mutational status of IPSS-M required genes. Median OS of patients in the 6 risk groups of the IPSS-M ranged from 192 months (prognosticated survival 125 months) in the very-low risk to 11 months (prognosticated survival 12 months) in the very high-risk group with prognostication becoming more precise in the higher risk groups beginning with the subgroup of moderate-low. Median OS of patients within the 5 risk groups of IPSS-R ranged from 137 months (prognosticated survival 106 months) in the very-low risk to 9 months (prognosticated survival 10 months) in the high-risk group with high precision of prognostication between low and very high-risk patients. Kaplan Meier based survival analysis of patients according to the different IPSS-M and IPSS-R cohorts is shown in Fig. 2. P-values were both < 0.001 with c-indices of IPSS-R and IPSS-M regarding OS of 0.60 and 0.68 respectively. Compared to subgroups of the IPSS-R, prognostication of AIPSS-MDS was most precise in the subgroup of intermediate and higher-risk patients and more precise than the IPSS-R when focusing on intermediate patients. Compared to the IPSS-M cohorts, prognostication of AIPSS-MDS was most precise in low, moderate-low and very-high risk groups and even more precise than the IPSS-M when looking at the subgroups low and very-high. A comparison of the predicted median OS according to AIPSS-MDS vs. IPSS-R vs. IPSS-M is shown in Table 3. Median Leukemia-free survival ranged from 162 months in the low risk to 15 months in the very high-risk group of IPSS-R and was not reached when looking at very low risk patients. Median LFS of the IPSS-M risk groups differed between 62 months in moderate low to 14 months in very high-risk patients and was not reached in patients of the very low and low risk groups. Regarding prediction precision of median LFS according to IPSS-R and IPSS-M, the IPSS-M appeared to be slightly more precise beginning with the group of moderate low patients while the IPSS-R was better regarding low-risk patients. Median LFS using Kaplan-Meier according to the different IPSS-M and IPSS-R cohorts is shown in Fig. 3. C-index of IPSS-R and IPSS-M regarding LFS was 0.6 and 0.69 respectively. A comparison of the predicted median LFS according to IPSS-R vs. IPSS-M is shown in Table 4. Kaplan-Meier based OS and LFS of patients divided into quintiles is shown in Fig. 4. Regarding Re-stratification of patients from IPSS-R to IPSS-M, there were more very low risk patients and less very high-risk patients according to IPSS-R than to IPSS-M (40 vs. 31 and 18 vs. 31) with a higher number of patients being up- than downgraded (35 vs. 15%). 98% of patients categorized as very low remained in the very-low and low risk group of IPSS-M. Some of the low-risk patients of the IPSS-R had an even lower risk according to IPSS-M while 37% were upstaged to higher risk groups. 36% of patients with high risk by IPSS-R were upgraded to the very-high risk cohort with 17% of patients being down staged respectively. Of the highest risk group according to IPSS-R, 17% were downgraded to minor risk groups. Re-stratification of patients from IPSS-R to IPSS-M is shown in Fig. 5.

**Table 3.** Median OS of patients compared to predicted OS by IPSS-M, IPSS-R and AIPSS-MDS

	Predicted median OS by IPSS-R	Observed median OS of patients	Predicted median OS by AIPSS
Very low (*n* = 40)	106	137	87
Low (*n* = 80)	64	69	57
Intermediate (*n* = 36)	36	40	40
High (*n* = 29)	19	34	17
Very high (*n* = 18)	10	9	10
	Predicted median OS by IPSS-M	Observed median OS of patients	Predicted median OS by AIPSS
Very low (*n* = 31)	125	192	87
Low (*n* = 72)	72	69	62
Moderate low (*n* = 34)	55	50	44
Moderate high (*n* = 21)	34	40	26
High (*n* = 18)	20	20	27
Very high (*n* = 31)	12	11	11

**Table 4:** Median LFS of patients compared to predicted LFS by IPSS-M and IPSS-R

	Predicted median LFS by IPSS-*R*		Observed median LFS of patients	
Very low (*n* = 40)	n.r.		n.r.	
Low (*n* = 80)	129,6		162	
Intermediate (*n* = 36)	38,4		62	
High (*n* = 29)	16,8		34	
Very high (*n* = 18)	8,8		15	
	Predicted median LFS by IPSS-M		Observed median LFS of patients	
Very low (*n* = 31)		116,4	n.r	
Low (*n* = 72)		70,8	n.r	
Moderate low (*n* = 34)		70,8	62	
Moderate high (*n* = 21)		27,6	47	
High (*n* = 18)		18	29	
Very high (*n* = 31)		9,1	14

## Discussion

Because myelodysplastic neoplasms comprise a group of heterogeneous diseases resulting in huge differences regarding treatment need, therapeutic options and overall survival, precise prognostication is of high importance for both patients and treating physicians. Therefore, the development of prognostication tools had its roots in the 1990 s where in 1997 an International MDS Risk Analysis Workshop combined and analyzed cytogenetic, morphologic, and clinical data from seven large, previously reported risk-based studies and introduced the International Prognostic Scoring System (IPSS) [7, 8]. 10 years later, the WHO classification-based prognostic scoring system (WPSS) dynamically classifying patients into five prognostic risk groups at any time during the course of the disease based on WHO subgroups, cytogenetics, and transfusion need was invented [9]. Another 5 years later, the IPSS was revised using 5 rather than 3 cytogenetic prognostic subgroups based on international data compiled by Schanz et al. [10], an adjusted marrow blast count and the depth of cytopenia herewith assigning patients to 5 rather than 4 prognostic categories now forming the IPSS-R [4]. Comparing the IPSS-R, WPSS and IPSS, best results regarding the ability to predict survival in MDS patients who received either BSC or induction chemotherapy or underwent allografting were obtained by the IPSS-R [11]. With the IPSS-M, the first model focusing on molecular genetics was introduced in 2022. Finally, the molecular profile of the underlying disease was integrated and lead to a more precise prognostication compared to the IPSS-R [12].

Due to the different circumstances and infrastructure of each country, molecular testing is still not routinely assessed resulting in a lower accuracy of IPSS-M prediction when one or more molecular features are missing [13]. Because of this fact, Sauta et al. considered a minimum data set of 15 relevant genes keeping the accuracy of IPSS-M prediction at 70 to 80%, while reducing the number of available genes to 10 or less resulted in a significantly lower accuracy of prediction [13]. Despite the fact that all mentioned prognostication tools have been validated multiple times, until today, there is no standard between different countries and even different treatment centers on the basis of which scoring system decisions regarding initiation and type of treatment should be made. To overcome the problem of complex and inaccessible molecular analyses, the AIPSS-MDS was introduced by the Spanish MDS group in 2023 [6]. Using 8 standard parameters in MDS patients and machine learning technique random survival forests, the AIPSS-MDS achieved superior accuracy in predicting OS and LFS in a Spanish patient cohort [6].

Our analyses aimed to validate and compare the IPSS-R, IPSS-M and AIPSS-MDS in the context of clinical daily practice using real world data, that have not been used for the development of the IPSS-R and the IPSS-M. Notably, out of more than 8500 patients of the Düsseldorf MDS Registry, only 5% could be included into our analyses due to missing or incomplete molecular data for the calculation of IPSS-M underlining the fact of fragmentary available molecular analyses. Based on data of 207 patients, we could show that prognostication of median overall survival taken as a whole was most precise by IPSS-M with a high accuracy in all risk groups except for very low risk patients. Prognostication of IPSS-R was also very precise but had its weakness in the very low risk and high-risk group. Comparing prognostication of AIPSS-MDS to the results of IPSS-R and IPSS-M, the AIPSS-MDS had its strength in intermediate and high-risk patients classified according to the IPSS-R as well as in moderate low, moderate high and very high-risk groups of the IPSS-M. When looking at leukemia-free survival, prognostication of the IPSS-M was again in total most precise with good precision starting from the moderate low group while prognostication of IPSS-R was less precise but better in low-risk patients.

We were not able to confirm the superiority of the AIPSS-MDS over the IPSS-R as described by the Spanish MDS group. Our results are also not in line with the data of Lincango et al., who showed greater prognostic power for OS with the IPSS-M and AIPSS-MDS than using the IPSS-R in patients of Argentina and Uruguay [14]. As the AIPSS-MDS is an AI-based prognostication tool trained and tested with a large cohort of Spanish patients, our patient population means a different patient population than it is used to. Furthermore, due to its AI-basis, it does not include expertise regarding clinical advancements like for example more appropriate Hb levels to subdivide patients and assign them into different groups. Belli et al. validated the AIPSS-MDS in a large cohort of patients from Latin-America also showing superiority of the AIPSS-MDS compared to the IPSS-R with even higher precision when excluding patients with CMML concluding a lower performance in this disease [15]. Controversially, Mosquera Orgueira et al. validated their prognostication tool in a lot of patients with CMML of the Spanish registry as well as a smaller Taiwanese patient cohort and showed accurate prediction of OS and LFS herewith highlighting the generalizability of their prognostic scoring system [16]. As our patient cohort did only include a very small number of patients with MP-CMML, we were unable to draw conclusions for this group based on our German and Austrian data.

In 2024, Mosquera Orgueira et al. tried to recalibrate their AIPSS-MDS model by integrating molecular data of up to 13 genes [17]. The results were quite surprising with only slight improvements of prognostication compared to the original model leading to the conclusion that clinical data in MDS patients remain of highest importance [17]. Regarding treatment options, the IPSS-M is most likely to shape future transplant decisions by identifying more patients – previously classified as low-risk by IPSS-R – as candidates for allografting.

Our study has some limitations, mainly due to the retrospective design and the small sample size of patients. As our analyses are retrospective and documentation of patients has not always been as extensive and disposable as today, we were not able to give evidence about all patient information. Since genetic analyses have evolved over the last 20 years and molecular testing has become more frequent, there is a huge lack of data. With the decision to exclude 1441 patients with incomplete molecular status, we may have increased and decreased the statistical power of our analyses at the same time preventing from vagueness of the IPSS-M (unknown/not assessed status of patients) while lowering the number of included patients that could have been fully prognosticated by IPSS-R and AIPSS-MDS. Nevertheless, regarding our German-Austrian cohort, all three tools demonstrated robust prediction of median OS and each has its merit. Among them, the IPSS-M provided the most accurate prediction of median OS while the frequent lack of molecular data remains an obstacle in daily clinical practice. Due to these circumstances, the IPSS-R remains the prognostication tool with the widest applicability. Based on our data, prognostication using the AIPSS-MDS is also feasible but less precise.

## Data Availability

The data that support the findings of this study are available from the corresponding author upon reasonable request.
